# Separated Type Atmospheric Pressure Plasma Microjets Array for Maskless Microscale Etching

**DOI:** 10.3390/mi8060173

**Published:** 2017-06-01

**Authors:** Yichuan Dai, Man Zhang, Qiang Li, Li Wen, Hai Wang, Jiaru Chu

**Affiliations:** 1 Department of Precision Machinery and Instrumentation, University of Science and Technology of China, Hefei 230026, China; daiyc@mail.ustc.edu.cn (Y.D.); Zm8218@mail.ustc.edu.cn (M.Z.); lqlqlq@mail.ustc.edu.cn (Q.L.); jrchu@ustc.edu.cn (J.C.); 2 School of Mechanical and Automotive Engineering, Anhui Polytechnic University, Wuhu 241000, China; wanghai@ahpu.edu.cn

**Keywords:** atmospheric pressure plasma microjets array, maskless etching, photoresist, MEMS, separation design, parallel processing

## Abstract

Maskless etching approaches such as microdischarges and atmospheric pressure plasma jets (APPJs) have been studied recently. Nonetheless, a simple, long lifetime, and efficient maskless etching method is still a challenge. In this work, a separated type maskless etching system based on atmospheric pressure He/O_2_ plasma jet and microfabricated Micro Electro Mechanical Systems (MEMS) nozzle have been developed with advantages of simple-structure, flexibility, and parallel processing capacity. The plasma was generated in the glass tube, forming the micron level plasma jet between the nozzle and the surface of polymer. The plasma microjet was capable of removing photoresist without masks since it contains oxygen reactive species verified by spectra measurement. The experimental results illustrated that different features of microholes etched by plasma microjet could be achieved by controlling the distance between the nozzle and the substrate, additive oxygen ratio, and etch time, the result of which is consistent with the analysis result of plasma spectra. In addition, a parallel etching process was also realized by plasma microjets array.

## 1. Introduction

In recent years, Micro Electro Mechanical Systems (MEMS) devices, especially micro-fluidic devices and bio-MEMS have attracted the attentions of researchers due to the significant potential in fields of biological processing and chemical reactions [1,2]. As one of the key processes for MEMS fabrication, plasma treatments have been widely used in various applications including etching [3], functionalization [4], and surface modification [5,6,7] owing to its cleanliness, reactivity, and high fabrication resolution based on photolithography [8]. Nonetheless, plasma processes have high operating costs and are time-consuming owing to their requirements of vacuum components and several steps of preparing the mask. Hence, an affordable, efficient method is urgently required of the localized plasma treatment. 

To solve these concerns, a large amount of effort has been made by researchers in recent decades. Microplasma generated by various types of microdischarges have been developed for the localized surface modification of materials [9,10,11,12,13]. For instance, microscale porous N_2_/C_2_H_2_ and He microdischarges array have been developed for the localized coating and functionalization of polymeric surface. This approach is known as “plasma printing” [10]; a 500 μm foldable paper-based microdischarges array has also been designed for flexible maskless patterning on the curved or structured surface [12,13]. These modification processes both require the substrate to be in contact with the microdischarges, which results in the resolution of tens to hundreds micrometers corresponding to the size of these devices. An agreement on microplasma devices is that they are suitable for modification of materials since their high electron density of 10^13^–10^16^ cm^−3^ [14] and large-scale localized treatment with dozens of microdischarges in array. However, the complex fabrication process of microdischarges and requirement of vacuum components makes these approaches time-consuming and expensive. On the other hand, non-thermal atmospheric pressure plasma microjet (APPμJs) have attracted a lot of attention due to their widespread applications in maskless materials processing. In the last decade, various types of He-O_2_/air APPμJs driven by radio frequency or low-frequency power have been developed for modification and removal of materials [15,16]. Compared with low-pressure microplasma devices such as microdischarges, APPμJs generate room-temperature plasma containing highly reactive species to downstream in open air, which gives them several advantages in direct treatment of hard and soft materials with outstanding flexibility and simplicity. For instance, a tapered air APPμJs produced by pulling quartz microcapillary has been developed for the removal of Parylene-C films [16,17]; in addition, Masaaki Nagatsu and co-workers have developed an APPμJ system based on ultrafine nanopipette nozzle for sub-micron modification and removal of carbon nanotubes and photoresists [18,19,20]. Their results are considered as the minimum line width of current APPμJs. Although APPμJs are a promising technology for fabrication polymer microstructures, careful consideration of the gas flow dynamics in plasma treatment is required because of their fragile thin tube wall and small tip [21]. Besides, continual collisions between energetic species and a tapered tube-wall [22] result in a requirement of higher ignition voltage in APPμJ systems. More importantly, parallel maskless processing remains a challenge for single microjet configurations of APPμJs. Therefore, improvement of the present maskless plasma fabrication approach is still needed. 

Here, we propose a novel maskless plasma process approach. In our system, a microfabricated micro/nano nozzle was attached to the outlet of millimeter scale capillary tube. When polymer films were exposed to the device at atmospheric pressure, downstream reactive species generated by plasma in the tube injected from micro/nano nozzle holes reacted with polymer films and led to the localized fabrication. Several advantages of our system were as follows: (1) ease and relatively low- cost MEMS technology were applied for fabrication of nozzle. Hence, dimensions of nozzle array could be micro/nanoscale based on precise microfabrication capacity, which satisfies the requirement of localized etching without mask; (2) plasma microjet array emanating from nozzle array makes high efficiency, large-area maskless etching realizable; (3) since we separate injection components from plasma generation components in our system, it is convenient to replace the nozzle if different size or number of nozzles in the array is needed, which would be conducive to the maintaining and updating the system; (4) since the system operated in ambient air, flexible scanning fabrication can be achieved while integrated with roll-to-roll systems. 

In this paper, the separated type atmospheric pressure plasma microjets array system was utilized for microscale polymer etching using a maskless and non-contact approach. Two main issues were investigated: (1) the electrical and spectra characteristic of the system which related to the capability for maskless etching; (2) the effect of the distance, additive oxygen ratio, and etch time between the nozzle and substrate on features of the microholes on polymer films. Finally, plasma microjets array etching process has also been studied.

## 2. Materials and Methods 

### 2.1. Main Components of Separated Type Atmospheric Pressure Plasma Microjets Array System

**Figure 1:**
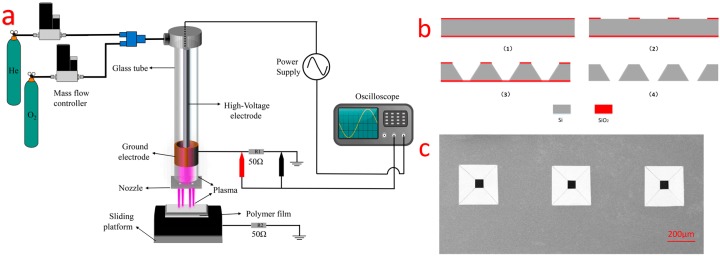
(**a**) The schematic diagram of the atmospheric pressure plasma microjets array system maskless etching process; (**b**) fabrication sequence of a Micro Electro Mechanical Systems (MEMS) nozzle; (**c**) scanning electron microscope (SEM) image of single hole on the nozzle, the upper and lower diameter of the hole were around 262 μm and 50 μm, respectively.

The schematic diagram of atmospheric pressure plasma microjets array system and its maskless etching process is shown in Figure 1a. This system included an APPJ based on the dielectric barrier discharge (DBD) principle, a micro nozzle holes array, a *Z*-axis sliding platform, an optical apparatus, and an analytical balance. A glass tube with 4 mm inner diameter (ID) and 6 mm outer diameter (OD) was used as the dielectric material in APPJ. The 9 mm long ring-shape copper ground electrode was placed around the glass tube 3 cm from the outlet. The high-voltage copper electrode was 3 mm in diameter and 7 cm in length, which was machined to fit tight around the glass tube. A polytetrafluoroethylene (PTFE) hollow cylinder 7 cm in length was wrapped around the glass tube. 

The atmospheric pressure plasma microjet was driven by a low frequency (around 11 kHz) sinusoidal high voltage source. Besides, a Tektronix P6015A (Tektronix, Beaverton, OR, USA) high voltage probe was applied to measure the voltage. The discharge current of plasma in the glass tube and plasma microjet was calculated by the voltage across a 50 Ω non-inductive resistor R1 and R2, respectively. The voltage and current waveforms were recorded by a Tektronix digital oscilloscope DPO-3014.

Moreover, a single-hole nozzle was used when measuring spectra characterization of atmospheric pressure plasma microjet, which avoids the measuring plasma microjets array interfered by other plasma microjets. To clarify the reactive species of plasma microjet in the open air, the emission spectra of the atmospheric pressure plasma microjet was measured using a fiber optical spectrometer (AvaSpec-ULS2048-2-USB2, Avantes, Apeldoorn, The Netherlands). The fiber integrated with the collimating lens was placed at the side of the microjet and collected optical emission from plasma.

As a preliminary component of our system, the micro nozzle holes array was fabricated on a 150 ± 10 μm thick silicon wafer by MEMS fabrication process, as shown in Figure 1b. Nozzle square holes for plasma injection were fabricated by wet etching based on a SiO_2_ layer mask formed by wet oxidation. As the result of anisotropic wet etching property of silicon, the pyramidal-structure cavities array with their upper and lower dimensions of 262 μm and 50 μm was formed, Figure 1c shows the SEM image of the micro nozzle array. Smaller dimensions of square nozzle arrays from several micrometers to hundreds of nanometers could also be fabricated by controlling fabrication parameters in future work.

Adjusting the distance and parallelism between the surface of nozzle and polymer sample was a must since the consistency of microstructures fabricated by the microjet should be guaranteed in parallel processing. An optical apparatus was built based on the equal-thickness interference principle. Two transparent and insulated polymethylmethacrylate (PMMA) plates applied as platforms of nozzle and polymer. The nozzle adhered to the lower surface of the nozzle platform while the substrate was placed on the *Z*-axis sliding platform. Interference fringes were formed as a result of laser beam being focused on two platforms. It was obvious that the wedge angle formed by two plates correlated to the number of interference fringes based on the equal-thickness interference principle. The wedge angle was about 1.5^−3^ degree after adjustment of micrometer screws on the nozzle plate, which satisfied the requirement for parallel plasma maskless processing.

**Figure 2:**
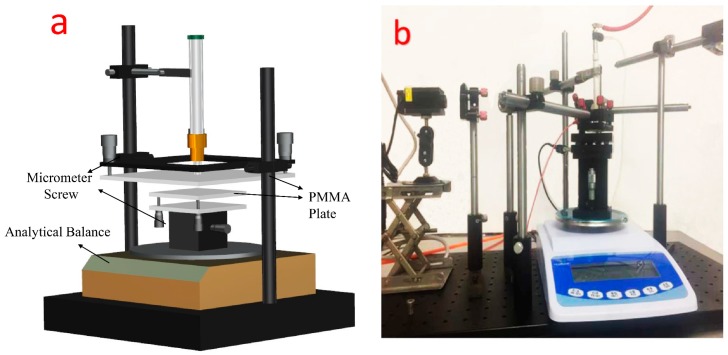
(**a**) The schematic of leveling device for the maskless parallel etching process. Optical apparatus was not shown in this schematic; (**b**) the separated type atmospheric pressure plasma microjets array system.

“Force feedback” method was applied for distance control. Operating procedures were that kept raising the Z sliding platform until contact occurs between nozzle and platform surface firstly, the digital readout from the balance vary from zero to certain value in that the nozzle gives a push force to the platform. Then we adjusted platform surface to the appropriate height which corresponds to expected distance between nozzle and platform. The schematic of the leveling device is shown in Figure 2a, the plasma microjet system is exhibited in Figure 2b.

### 2.2. Etching and Characterization

Photoresist is a light-sensitive, soft polymer material that used in processes such as photolithography. Photoresist etching by oxygen plasma is one of the most widely in MEMS and LSI fabrication technology [23], in that a mass of reactive oxygen species (ROS) such as oxygen atoms generated by plasma could react with polymer films, then form volatile small molecules and thus remove of material [24,25]. To investigate the plasma maskless etching capacity of our device on photoresist, the 7.5 μm thickness AR-3210 positive photoresist was used in this study which was spin-coated on a slide-glass substrate. The etching time varied from 1 min to 5 min. The effect of distance between the nozzle and the substrate, the additive oxygen ratio was investigated. Furthermore, the 2D morphologies of microholes on photoresist etched by single plasma microjet were measured by a scanning electron microscope (SEM) and an optical microscope. Additionally, the stylus profiler was applied for measuring the irregular depth shape of the etched microholes.

## 3. Results and Discussions 

### 3.1. The Electrical Characteristics of the Atmospheric Pressure Plasma Microjet

**Figure 3:**
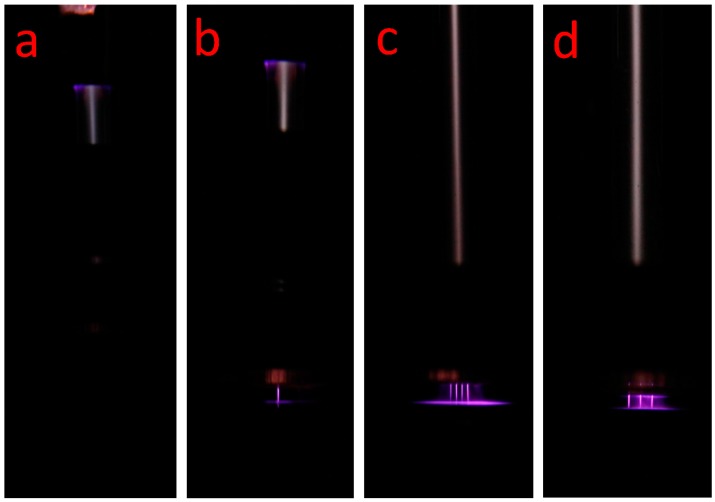
(**a**) Optical image of single plasma microjet without substrate and sliding platform no plasma plumes was seen by naked eyes; (**b**,**c**) optical image of single plasma microjet and 1 × 3 and 2 × 2 plasma microjets array in the dark condition; (**d**) discharge generated surround the 2 × 2 plasma microjets when the distance was close to 1 mm. Experiment conditions: applied voltage was 4.5 kV_pp_, 11 kHz; 700 sccm helium mixed with 7 sccm oxygen.

Helium/oxygen plasma was easily generated in the glass tube when the voltage raised to 3.5 kV_pp_. After raising applied voltage up to about 4.5 kV_pp_, plasma microjet expanded out of the glass tube was still too short to be visible to the naked eyes, as shown in Figure 3a. A brighter and longer plasma microjet was observed when placed the Z sliding platform connected to electrical ground and the substrate under the nozzle, as shown in Figure 3b. This was due to the substrate acting as a floating electrode, the plasma microjet was greatly enhanced when contact with the platform. The plasma in the glass tube increased the density of the seed electrons to lower the ignition voltage of plasma microjet, which resulted in a non-thermal and stable plasma microjet [26]. The plasma microjet array was formed successfully in Figure 3c. In addition, silicon micro-nozzles get charged from the bombardment of plasma in the glass tube, the unexpected plasma was generated by the strong electric field which exceeds the threshold for ignition in the whole space under the nozzle, as shown in Figure 3d.

**Figure 4:**
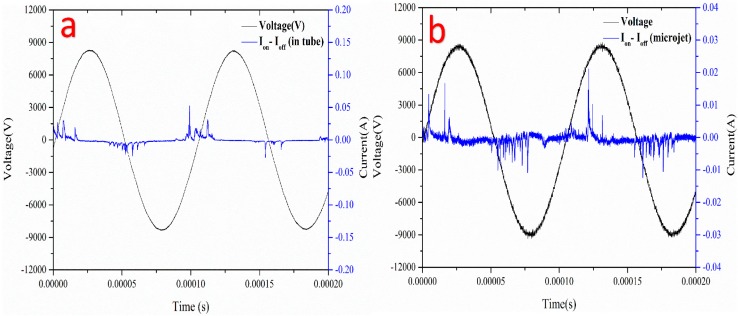
(**a**,**b**) Measured waveforms of applied voltage and discharge current of dielectric barrier discharge plasma in the glass tube and single plasma microjet, respectively. Experiment conditions: helium/oxygen mixed flow rate of 700 sccm/7 sccm, the frequency was around 11 kHz.

As shown in Figure 4a,b, the discharge current waveform was measured by subtracting the displacement current from the total current, corresponding the situation in Figure 3a,b. Plasma microjet could hardly be seen in Figure 3a, thus, discharge waveforms in Figure 4a mainly represent the DBD discharge current in the glass tube. The peak value of discharge current was around 50 mA, which suggested that the plasma was active. According to the literature [27], two discharge current pulses were commonly observed in one DBD discharge cycle. It is speculated that other herringbone-like current peaks in Figure 4a may represent the discharge between charged silicon nozzle and the high-voltage electrode. 

Besides, the current in Figure 4b represents the discharge current of plasma microjet. An asymmetric characteristic of discharge current was observed in Figure 4b, as the amplitude of discharge current in positive polarity was relatively larger than in negative polarity. Some speculations for the formation of plasma jet may explain this phenomenon. It is known that plasma jet of this kind propagates by a streamer mechanism, in which a source of electrons is needed to form the ionization front [28]. The electrons in the negative plasma streamer are scattered, in that an enhanced electric field could not be created in the plasma channel to maintain the streamer propagation, as compared to the positive plasma streamer [29].

### 3.2. The Spectral Characteristics of Atmospheric Pressure Plasma Microjet

Since different additive oxygen ratio of working gas affected the O-containing radicals in plasma, the effect of additive oxygen gas on the emission intensities from plasma microjet should be investigated. The helium gas was maintained at a flow rate of 700 sccm, the oxygen gas flow rates varied from 0 sccm to 115 sccm.

**Figure 5:**
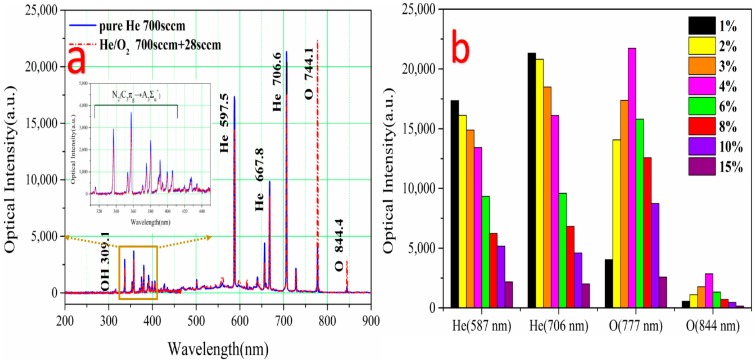
(**a**) Optical emission spectrum (OES) of the plasma microjet from 200 nm to 900 nm measured in the pure helium and helium/oxygen plasma, respectively, the applied voltage was 8.5 kV_pp_, 11 kHz. The inner picture was the magnified details of OES from 300 nm to 450 nm; (**b**) comparisons of the change of the emission intensities from He (587 nm and 706 nm) and O radicals (777 nm and 844 nm) in different additive oxygen ratio increasing from 0 to 15%.

Figure 5a shows a comparison of the optical emission spectrum (OES) observed in pure helium and He/O_2_ mixed plasma microjet. It was illustrated that ultraviolet (UV) radiation attributed to N-containing radicals at wavelengths of 300–410 nm in OES, OH band, and atomic oxygen lines were detected at the wavelength of 309, 777, and 844 nm, respectively. N-containing radicals such as N_2_ band and OH band was generated by the electrons collide with helium radical species, nitrogen components, and vapor in ambient air [30,31]. It was noted that the addition of oxygen to the He plasma caused a decrease in the optical intensity of N-containing radicals while the intensity of reactive oxygen species (ROS) increased obviously. This suggested that some electrons are consumed to produce O radicals. Thus, collisions with air molecules decreased [32], the intensity of N_2_ band and OH band decreased slightly.

As shown in Figure 5b, different additive oxygen ratio affected the intensity of species in the range from 0 to 15%. The intensities of He were decreased with additive oxygen flow. Furthermore, highest intensity of ROS could be obtained at the additive oxygen ratio of 4%. This phenomenon was consistent with the reduction of ROS at the ratio above 4% may not only be caused by decreased plasma intensity but also the reaction of O + O_2_ + He → O_3_ + He that producing ozone [33]. The O_3_ production became more effective at the higher O_2_ ratio, thus the intensity of ROS may also decrease at high O_2_ concentrations. In addition, the plasma microjet quenched gradually when oxygen flow raised to 126 sccm (18%). Correspondingly, the intensity of all species significantly decreased to almost 0.

### 3.3. The Maskless Etching of Photoresist 

#### 3.3.1. Maskless Etching of Photoresist Using Single-Hole Plasma Microjet

A single-hole nozzle was used to quantify the result of maskless etching of photoresist film initially. Plasma was ignited by He/O_2_ mixed gas with different additive oxygen ratio and 700 sccm helium. The applied voltage was maintained at 8.5 kV_pp_ after the generation of plasma microjet. 

**Figure 6:**
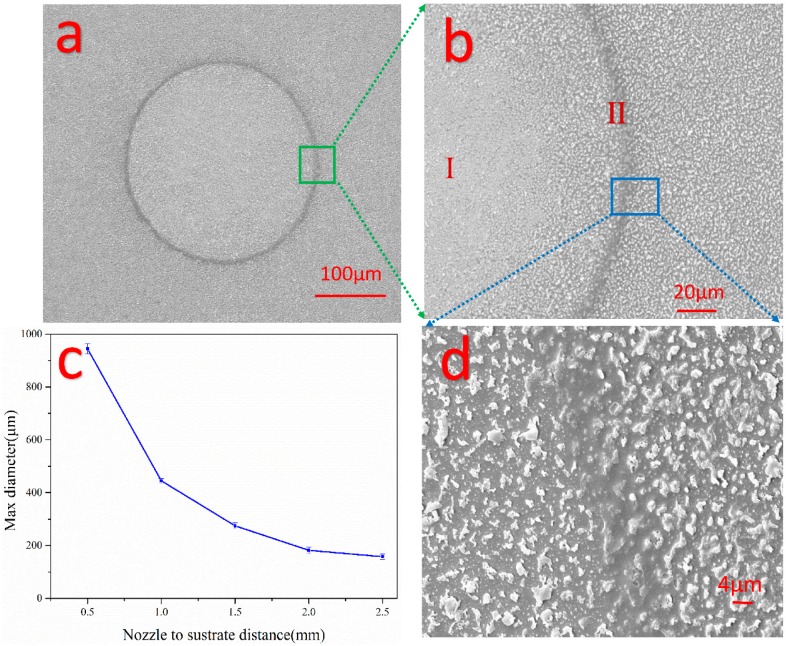
(**a**) SEM image of microholes etched on the photoresist by plasma microjet. The diameter of the holes was 266.7 μm. The etch parameters were: distance between nozzle and polymer surface was 1.5 mm, the etch time was 3 min; (**b**) magnified details of side wall of the microhole in the green box; (**c**) the maximum diameter of the microholes on polymer at different distance between nozzle and substrate; Experimental conditions: 700 sccm helium gas and 28 sccm additive oxygen at applied voltage of 8.5 kV_pp_, frequency was around 11 kHz; (**d**) magnified SEM image of small area formed by sputtering effect of plasma microjet in the blue box.

The effect of the distance between the nozzle and the polymer surface was examined firstly. Figure 6a shows SEM images of the microholes on the photoresist etched by plasma microjet. Additionally, Figure 6b illustrates magnified SEM image on the side wall of the microhole fabricated by plasma microjet. Still, some residual photoresist particles remained in the etched region I. Furthermore, the surface on the outside of microholes was decorated with the debris, of which the size was varied from nanometers to micrometers, as shown in Figure 6d. The nonhomogeneous etched region II was owing to the expansion effect of the plasma microjet coming into contact with ambient air. The data in Figure 6c indicated that the maximum diameter of microholes was etched at the closest distance of 0.5 mm, smaller microholes were etched as distance increased. Possible explanations are drawn that when plasma microjet comes into contact with the substrate, ROS will diffuse and move along the surface via gas pressure. Hence, the closer the distance was, the bigger diffusion area it formed, which leads to a bigger etched microhole. Moreover, highly applied voltage and closer distance enhanced the electric field between the silicon and the substrate. Thus, an active plasma region was generated surrounding the plasma microjet, which resulted in an enlarged etched area. Hence, much bigger microholes were etched when the distance was closer than 1 mm.

In addition, the experiments show that circle-shaped microholes of several hundred micrometers were etched by plasma microjet emanating from 50 μm square nozzle. This might be caused by the enlarged plasma zone while the plasma microjet reached the polymer and formed a larger disk-like area on its surface compared with the dimension of plasma microjet. This plasma expansion typically existed in DBDs due to the spreading of the accumulated discharge [34] and movement of reactive species above the polymer surface. We note here that an understanding of the exact dimension of the plasma microjet would be greatly enhanced by imaging of plasma bullet. However, at present, such facilities are unavailable and will form part of our future experimental work on the system.

**Figure 7:**
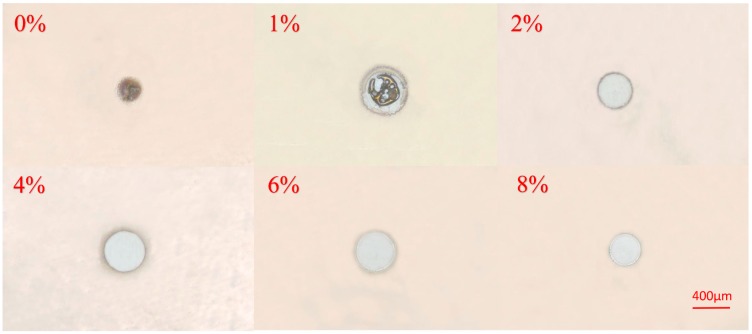
Optical microscopy images of photoresist etched by plasma microjets with different additive oxygen ratio from of 0, 1, 2, 4, 6, and 8%, the diameters from 2 to 8% were 322, 438, 429, and 310 μm, respectively. Other etch parameters were: applied voltage 8.5 kV_pp_ with a frequency of 11 kHz, helium flow rate fixed at 700 sccm, etch time of 3 min and a distance of 1 mm.

Another important process parameter affecting the diameter of microholes was additive oxygen ratio. Figure 7 shows optical images of etching results in different additive oxygen ratio. It was illustrated that the maximum diameter of microholes was fabricated at the additive oxygen ratio of 4% since the highest intensity of ROS acted as a key reactive species of removing photoresist. It was seen that with the decrease of oxygen flow rate, high temperature, and large amount of high energetic reactive species in the center of the plasma jet accelerated the carbonization and formed a nonhomogeneous and rough zone in the center of microholes [35]. Additionally, as the additive oxygen ratio increased from 0 to 8%, the diminution of rough and nonhomogeneous etching boundary of etched microholes was seen in Figure 7. This could be explained by that while oxygen admixture produced chemically active species, the physical sputtering delivered nearly no contribution to etching process subsequently [36]. The additive oxygen acted as electronegative gas, causing the reduction of metastable He, thus eventually weakening the physical sputtering and decreasing the amounts of sputtering fragments at the boundary of microholes [37].

**Figure 8:**
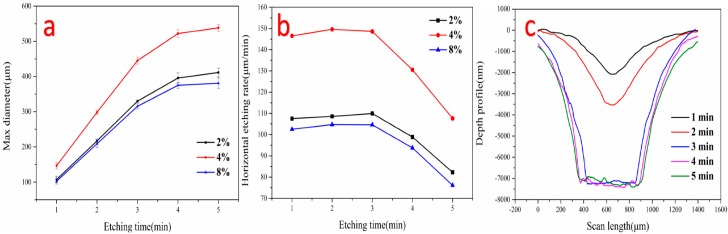
(**a**) The maximum diameter of the microholes on the polymer at the different distance between nozzle and substrate; (**b**) time-dependent maximum diameter of the microholes at different additive oxygen ratios; (**c**) depth profiles of microholes etched by plasma microjets. Experimental conditions: 700 sccm helium gas and 28 sccm additive oxygen at an applied voltage of 8.5 kV_pp_, the frequency was around 11 kHz.

Different etch features can also be fabricated by adjusting the etching time. As shown in Figure 8a, the data of the diameter of microholes varied by etching time under different additive oxygen ratio was shown in Figure 8a. Figure 8b indicated that as the etch time increase, the horizontal etching rate of photoresist increased slightly in the first 3 min, and decreased significantly in subsequent. Indeed, this notable feature was attributed to the weaker reactivity of plasma microjet away from the central axis. Moreover, high intensity of ROS resulted in a stronger etching capability of the plasma microjet, which caused higher etching rate in horizontal of microholes compared the etch result of 2 to 4% and 8%. It is expected that increasing of the etching rate could also be achieved by using higher applied voltage and higher oxygen flow rate [35].

For the purpose of evaluating the morphologies of etching results, the depth profiles were measured by stylus profile crossing the center of the microholes. Figure 8c shows the depth profile of microholes etched in photoresist films with etching times from 1 min to 5 min. It was founded that a V-shape depth profile was measured before 2 min and platform was formed after treated 3 min. This indicated that all photoresist was removed in the treatment area. It could be calculated that the highest etching rate was approximately 4 μm/min. As the etch time increased after 3 min, the profile changed slightly. The asymmetries of these profiles may originate from the glass tubes was not exactly perpendicular to the polymer surface.

#### 3.3.2. Maskless Etching of Photoresist Using Array-Holes Plasma Microjet

Based on the above fundamental etch characteristics of the plasma microjet formed by the single-hole nozzle, parallel etching process could be achieved by controlling the distance, additive oxygen ratio, and the etch time using the micro nozzle array, as shown in Figure 3c.

As a key issue for large-scale applications of the plasma jet array, the spatial uniformity of plasma microjets array caused deviation in diameter of each microholes, which reduces the quality of maskless fabrication. The spatial nonuniformity of the plasma microjet array has been investigated and it is caused by the inhomogeneous distribution of the electric and flow field [30] and the repulsion of adjacent microjet caused by momentum coupling and Coulomb force [36]. 

**Figure 9:**
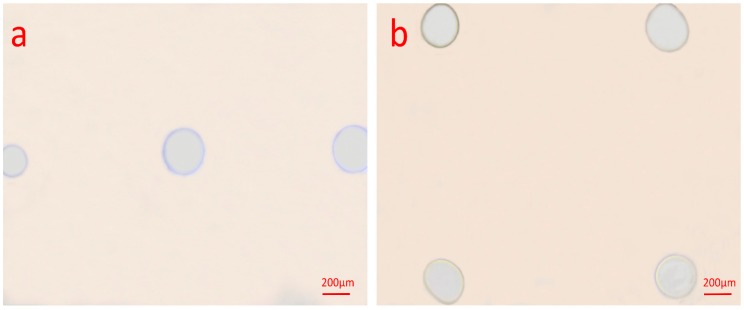
(**a**,**b**) Optical microscopy images of photoresist etched by plasma microjets array of 1 × 3 and 2 × 2. The diameter of the microholes from left to right in (**a**) was 196, 251, and 266 μm; diameters in (**b**) were 251, 268, 253, and 258 μm. Experiment conditions: 700 sccm helium gas and 28 sccm additive oxygen at an applied voltage of 7.5 kV_pp_ and 8.5 kV_pp_, the frequency was around 11 kHz, the distance was 1 mm.

In our experiment, an array of 1 × 3 microjets was generated by corresponding nozzle hole array, the distance between adjacent nozzle was 1 mm, as shown in Figure 9a. Nonuniformity of 2 × 2 etched microholes array was also obtained when using nozzle holes array with their spacing of 0.5 mm and 0.8 mm. However, similar size of microholes was etched if increasing the spacing to 1.5 mm, as can be seen the 2 × 2 array in Figure 9b. With the increase of the spacing and symmetric position of nozzle holes along the high-voltage electrode, the electric field of the adjacent nozzle was relatively homogeneous, which suggests that the consistent excitation of the adjacent plasma streamer. Moreover, higher applied voltage was considered as an increasing of the electric field in each plasma plume of the 2 × 2 array. The inhomogeneity of the plasma microjets array was less once the electric field exceeds the threshold for igniting the plasma microjet [38]. As a result, the diameter of each microhole in the 2 × 2 array is almost the same. However, the shape of each microhole in both arrays was a defective circle-shape, which suggests the inhomogeneous distribution of reactive species in the etched region. There is a speculation that, as the distance between the nozzle and the center axis of high-voltage electrode increased, the electric field was weakened rapidly at the edge position of the nozzle. According to the literature [39], the structure of multi-electrodes structure could improve the uniformity of plasma microjet array via the homogeneous electric field distribution. In the future, the electrode structure of an atmospheric pressure plasma microjet should be optimized based on theory and simulation.

## 4. Conclusions

In this study, a novel and simple system based on the atmospheric pressure plasma jet and microfabricated MEMS nozzle has been investigated for maskless etching of photoresist films. The plasma microjet was emanated from the nozzle, which could etch a single microhole on the photoresist successfully. The highest etching rate of the AR-3210 photoresist film reached 4 μm/min. The morphology of microholes affected by the distance between the nozzle and substrate, additive oxygen ratio, and etching time were investigated. Spectra characteristics of plasma microjet verified the etching results that the maximum diameter was obtained at an O_2_ admixture of 4%, lower additive oxygen flow rates led to a carbonization phenomenon. Additionally, parallel plasma processing has been realized via nozzle array. However, an improvement in the consistency of etched microholes array is still required, comprehensive investigation of the parallel etching properties affected by gas flow rate and distribution of nozzle holes array is also needed. Further research will be aimed at these targets. 

Based on this preliminary experiment, it could be expected that large-scale flexible manufacturing could be realized using the system integrated with roll-to-roll system soon. This maskless fabrication method using the atmospheric pressure plasma jet and MEMS nozzle has potential applications in localized fabrication of micropatterns and localized surface modification of several kinds of materials.
